# Responsiveness of Candidate Genes on *CoPv01^CDRK^/PhgPv01^CD^^RK^* Loci in Common Bean Challenged by Anthracnose and Angular Leaf Spot Pathogens

**DOI:** 10.3390/ijms242216023

**Published:** 2023-11-07

**Authors:** Maike Lovatto, Maria Celeste Gonçalves-Vidigal, Mariana Vaz Bisneta, Alexandre Catto Calvi, Josmar Mazucheli, Pedro Soares Vidigal Filho, Eduardo Gomes Rosa Miranda, Maeli Melotto

**Affiliations:** 1 Departamento de Agronomia, Universidade Estadual de Maringá, Maringá 87020-900, Brazil; 2 Departamento de Estatística, Universidade Estadual de Maringá, Maringá 87020-900, Brazil; 3 Department of Plant Sciences, University of California, Davis, CA 95616, USA

**Keywords:** *CoPv01^CDRK^/PhgPv01^CDRK^* loci, candidate gene expression, common bean–anthracnose interaction, common bean–angular leaf spot interaction, plant defense genes

## Abstract

Anthracnose (ANT) and angular leaf spot (ALS) are significant diseases in common bean, leading to considerable yield losses under specific environmental conditions. The California Dark Red Kidney (CDRK) bean cultivar is known for its resistance to multiple races of both pathogens. Previous studies have identified the *CoPv01^CDRK^/PhgPv01^CDRK^* resistance loci on chromosome Pv01. Here, we evaluated the expression levels of ten candidate genes near the *CoPv01^CDRK^/PhgPv01^CDRK^* loci and plant defense genes using quantitative real-time PCR in CDRK cultivar inoculated with races 73 of *Colletotrichum lindemuthianum* and 63-39 of *Pseudocercospora griseola*. Gene expression analysis revealed that the *Phvul.001G246300* gene exhibited the most elevated levels, showing remarkable 7.8-fold and 8.5-fold increases for ANT and ALS, respectively. The *Phvul.001G246300* gene encodes an abscisic acid (ABA) receptor with pyrabactin resistance, PYR1-like (PYL) protein, which plays a central role in the crosstalk between ABA and jasmonic acid responses. Interestingly, our results also showed that the other defense genes were initially activated. These findings provide critical insights into the molecular mechanisms underlying plant defense against these diseases and could contribute to the development of more effective disease management strategies in the future.

## 1. Introduction

*Colletotrichum lindemuthianum* (Sacc. & Magnus) Briosi & Cavara is a highly destructive pathogen that causes anthracnose (ANT) in common beans. In conditions of low temperature and high humidity, ANT can result in up to 100% yield losses. Furthermore, the pathogenic variability of *C. lindemuthianum* and the emergence of new races have resulted in the reduction or total loss of yield of previously resistant cultivars [1]. Angular leaf spot (ALS) is another disease that impacts common beans globally, caused by *Pseudocercospora griseola* (Sacc.) Crous & U. Braun. This disease can lead to yield losses of up to 70% [2,3,4]. Adopting genetically resistant cultivars provides a cost-effective, user-friendly, and environmentally conscious strategy for managing *C. lindemuthianum* and *P. griseola* infections in common beans [4]. Consequently, identifying and molecularly characterizing resistance genes is crucial for enhancing resistance efficacy and durability [5,6,7,8].

The *Co* and *Phg* loci associated with ANT and ALS, respectively, are often found in disease resistance clusters on various chromosomes. Although several independent genes confer resistance to *C. lindemuthianum*, most resistance genes found in Andean cultivars have been mapped to the common bean chromosome Pv01. The *Co-1*, *Co-1^2^*, *Co-1^3^*, *Co-1^4^*, *Co-1^5^,* and *Co-1^HY^* alleles of the *Co-1* genes are present in the cultivars of Michigan Dark Red Kidney (MDRK), Kaboon, Perry Marrow, AND 277, Widusa, and Hongyundou, respectively [9,10,11,12]. Other resistance genes were mapped to the end of Pv01: *Co-x* in Jalo EEP558 and *Co-AC* in Amendoim Cavalo [13,14]. Moreover, the *CoPv01^CDRK^/PhgPv01^CDRK^* gene in CDRK, which confers resistance to *C. lindemuthianum* races 73, 2047, and 3481, as well as race 63-39 of *P. griseola*, was recently fine-mapped to Pv01 [15].

A single dominant resistance gene primarily confers resistance to the ALS pathogen; however, recent studies have also identified quantitative resistance loci (QRLs) [16,17]. To date, five resistance loci have been mapped, including three independent loci, *Phg-1*, *Phg-2*, and *Phg-3*, located on chromosomes Pv01, Pv08, and Pv04, respectively [9,18,19]. Additionally, two major QRLs, *Phg-4* and *Phg-5*, have been found on Pv04 and Pv10 [16,17,20,21].

Candidate genes for ANT resistance loci have been analyzed through gene expression analysis to infer functionality in resistant cultivars [11,22,23]. Generally, examining the expression of the candidate and disease resistance genes can reveal their roles and interactions, thereby contributing to our understanding of how these genes collaborate in effective resistance responses. Chen [11] evaluated the expression analysis of four candidate genes at the *Co-1^HY^* allele in the Hongyundou cultivar inoculated with *C. lindemuthianum* race 81. The authors observed significant induction of all genes at an early stage. However, expression levels decreased at 24 h post-inoculation (hpi) and beyond. In the susceptible cultivar, high expression was only observed at 120 hpi, suggesting that delayed gene expression might facilitate pathogen penetration and proliferation, ultimately leading to disease development.

To elucidate the precise timing and magnitude of expression related to resistance against *C. lindemuthianum* race 73, Mahiya-Farooq [22] analyzed the expression of four candidate genes within the *Co-1* locus using near-isogenic lines that differ in the presence of the *Co-1^2^* resistance allele. They observed that the *Phvul.001G243800* gene exhibited substantially higher expression levels, nearly 144-fold, in the resistant near-isogenic line. The molecular basis of the ANT resistance *Co-x* locus was established by sequencing a 58-kb target region in the Jalo EEP558 cultivar. The *KTR2/3* gene was identified as an additional gene within a CRINKLY4 kinase cluster between the candidate genes *Phvul.001G243600* and *Phvul.001G243700*. Gene expression analysis demonstrated that *KTR2/3* was upregulated in Jalo EEP558 at 24 hpi in plants inoculated with strain 100 of *C. lindemuthianum* [13,23]. In summary, gene expression studies regarding the ANT resistance locus have identified the candidate genes *Phvul.001G243800* for *Co-1^2^, KTR2/3* for *Co-x* and *Phvul.001G243600* and *Phvul.001G243700* for *Co-1^HY^*, which showed high expression levels under inoculation conditions [11,13,22,23].

*Phaseolus vulgaris* CDRK from the breeding program at the University of California Davis is a landrace collected around Sacramento, CA, USA [24]. CDRK is resistant to Andean races 2, 39, and 55 and Mesoamerican races 9, 64, 65, 73, 89, 1545, and 2047, and race 3481 of *C. lindemuthianum* [15]. Allelism test revealed that *CoPv01^CDRK^*/*PhgPv01^CDRK^* gene is not allelic to *Co-1* [15]. Through fine mapping, *CoPv01^CDRK^*/*PhgPv01^CDRK^* was delimited in a genomic region of 33 Kb on chromosome Pv01, wherein the physical distances between *CoPv01^CDRK^/PhgPv01^CDRK^* and *Co-1*, *Co-x*, and *Co-1^HY^* are 211 kb, 193 kb, and 181 kb, respectively. This previous study detected five candidate genes: *Phvul.001G246000* (ATP-dependent RNA helicase), *Phvul.001G246100* (cation-dependent mannose-6-phosphate receptor), *Phvul.001G246200* (protein trichome birefringence-like 33), *Phvul.001G246300* (abscisic acid (ABA) receptor PYL5), and *Phvul.001G246400* (SNF2 domain-containing protein class 1-related). Additionally, the candidate genes *Phvul.001G245300* and *Phvul.001G246800*, which encode putative leucine-rich repeat protein kinases, are close to the *CoPv01^CDRK^*/*PhgPv01^CDRK^* loci [15].

The present study hypothesizes that each of the candidate genes that overlap with the *CoPv01^CDRK^/PhgPv01^CDRK^* loci on Pv01 exhibits distinct expression patterns in response to inoculations with race 73 of *C. lindemuthianum* and race 63-39 of *P. griseola* in the California Dark Red Kidney cultivar. The objective of this study was to investigate the expression patterns of the *CoPv01^CDRK^/PhgPv01^CDRK^* candidate genes (*Phvul.001G246000*, *Phvul.001G246100*, *Phvul.001G246200*, *Phvul.001G246300*, and *Phvul.001G245300*) in the CDRK cultivar, not only in response to *C. lindemuthianum* race 73 but also to *P. griseola* race 63-39, using gene expression analysis employing quantitative real-time PCR. Specifically, we seek to gain insights into their potential roles in the plant’s defense mechanisms against these pathogens, contributing to a deeper understanding of disease resistance in common beans.

## 2. Results

### 2.1. Phenotypic Evaluation of Cultivars

**Figure 1:**
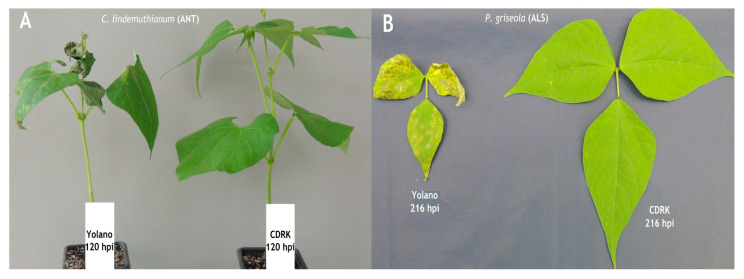
(**A**) Disease reaction of susceptible Yolano cultivar and no reaction in resistant California Dark Red Kidney cultivar at 120 h post-inoculation (hpi) with *C. lindemuthianum* race 73. (**B**) Disease reaction of susceptible Yolano cultivar and no reaction in resistant California Dark Red Kidney cultivar at 216 h post-inoculation with *P. griseola* race 63-39.

The results of inoculation of *C. lindemuthianum* race 73 at 120 h post-inoculation (hpi) onto the resistant cultivar CDRK and also the susceptible control cultivar Yolano are displayed in Figure 1. The disease in Yolano appears as small water-soaked lesions on the underside of the leaf and small sunken lesions on the stem and eventually leads to plant death. In contrast, no symptoms or hypersensitive response was observed in the resistant cultivar CDRK. Inoculations results of *Pseudocercospora griseola* race 63-39 onto CDRK resistant cultivar and Yolano susceptible cultivar 216 hpi are also displayed in Figure 1. The symptoms observed in Yolano cultivar were angular lesions in leaf area leading to early defoliation and plant death.

### 2.2. Differential Expression of Candidate and Defense Genes in the CDRK Cultivar Inoculated with Race 73 of C. lindemuthianum

We conducted an in-depth exploration into the expression patterns of the following candidate genes: *KTR2/3*, *Phvul.001G243800*, *Phvul.001G244300*, *Phvul.001G244400*, *Phvul.001G244500*, *Phvul.001G245300*, *Phvul.001G246000*, *Phvul.001G246100*, *Phvul.001G246200*, and *Phvul.001G246300*. These genes are located in regions that overlap with *Co-x*, *Co-1^2^*, *Co-AC*, and *CoPv01^CDRK^/PhgPv01^CDRK^* on the Pv01 chromosome. Additionally, defense genes *PR1a*, *PR1b*, and *PR2*, were evaluated to identify the molecular basis of CDRK resistance upon *C. lindemuthianum* race 73 inoculation.

**Figure 2:**
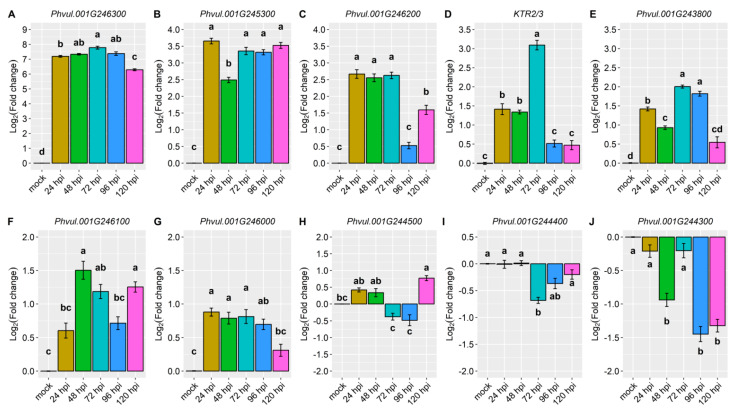
Relative expression of candidate genes: (**A**) *Phvul.001G246300*; (**B**) *Phvul.001G245300*; (**C**) *Phvul.001G246200*; (**D**) *KTR2/3*; (**E**) *Phvul.001G243800*; (**F**) *Phvul.001G246000*; (**G**) Phvul.001G246100; (**H**) *Phvul.001G244500*; (**I**) *Phvul.001G244400*; and (**J**) *Phvul.001G244300* in California Dark Red Kidney at 24, 48, 72, 96, and 120 h post-inoculation (hpi) with race 73 of *C. lindemuthianum* and a mock. The results are presented as logarithmic base 2 of the fold change of gene expression. Means with the same letter, for each gene, are not significantly different at the 5% significance level, using the Alexander-Govern test.

**Figure 3:**
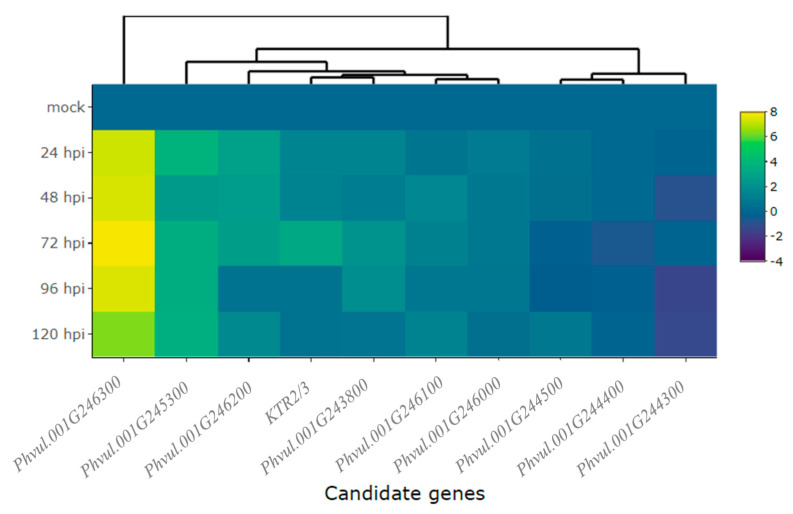
Heatmap of relative expression of candidate genes for the *CoPv01^CDRK^/PhgPv01^CDRK^* and genes proximal to these loci in California Dark Red Kidney at 24, 48, 72, 96, and 120 h post-inoculation (hpi) with race 73 of *C. lindemuthianum* and a mock. The genes evaluated were *Phvul.001G246300*, *Phvul.001G245300*, *Phvul.001G246200*, *KTR2/3*, *Phvul.001G243800*, *Phvul.001G246100*, *Phvul.001G246000*, *Phvul.001G244500*, *Phvul.001G244400*, and *Phvul.001G244300*. Yellow shading indicates higher expression and dark blue shading lower expression.

Detailed information about the evaluated genes, accompanied by their functional annotations, can be found in Table S1. The magnitude of gene expression levels within the candidate and defense genes are shown in Table 1. Particularly, the gene *Phvul.001G246300* standout among all the candidate genes in the CDRK cultivar. This gene exhibited a remarkable 7.2-fold change in expression at 24 hpi in the resistant CDRK cultivar. As the interaction progressed to 72 hpi, the gene *Phvul.001G246300* showed significantly heightened expression of 7.8-fold. Moreover, the expression of *Phvul.001G246300* remained constant across multiple time points: 24, 48, 72, 96, and 120 hpi (Figure 2A and Figure 3, and Table 1).

Expression of the candidate gene *Phvul.001G245300* revealed an approximate 3.7-fold increase at 24, 72, 96, and 120 hpi (Figure 2B), and *Phvul.001G246200* expression increased 2.7-fold at 24, 48, and 72 hpi (Figure 2C and Table 1). KTR2/3 expression exhibits higher expression only at 72 hpi, a 3.1-fold change (Figure 2D and Table 1). Although these genes exhibited some different expression levels in relation to the mock, they did not show large differences, as observed in *Phvul.001G246300* or in pathogen-related genes (Figure 2 and Figure 3, and Table 1).

The candidate genes *Phvul.001G243800*, *Phvul.001G246100,* and *Phvul.001G246000* had the lowest levels of relative expression. The *Phvul.001G243800* gene exhibited 2.0-fold change in relative expression at 72 and 96 hpi (Figure 2E and Table 1). *Phvul.001G246100* and *Phvul.001G246000* displayed an average of 1-fold change in relative expression (Figure 2F,G and Table 1). The candidate genes for resistance in Amendoim Cavalo, namely, *Phvul.001G244500*, *Phvul.001G244400*, and *Phvul.001G244300*, were not expressed in CDRK plants after inoculation of race 73 in relation to mock plants, indicating that these genes may not be involved in the *CoPv01^CDRK^/PhgPv01^CDRK^* response to *C. lindemuthianum* race 73 (Figure 2H–J and Figure 3, and Table 1).

**Figure 4:**
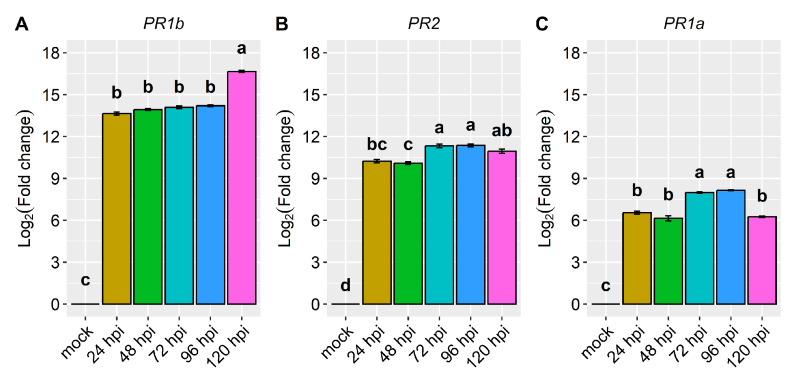
Relative expression of plant defense genes: (**A**) *Phvul.006G196900* (*PR1b*); (**B**) *Phvul.009G256400 (PR2);* and (**C**) *Phvul.003G109100* (*PR1a*) in the common bean cultivar of California Dark Red Kidney at 24, 48, 72, 96, and 120 h post-inoculation (hpi) with race 73 of *C. lindemuthianum* and a mock. The results are presented as logarithmic base 2 of the fold change of gene expression. Means with the same letter, for each gene, are not significantly different at the 5% significance level, using the Alexander-Govern test.

**Figure 5:**
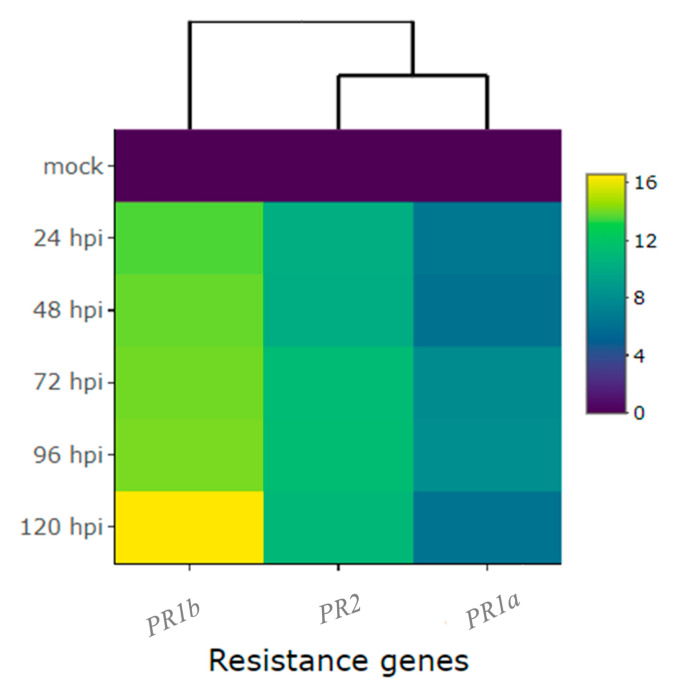
Heatmap of relative expression of plant defense genes *Phvul.006G196900* (*PR1b*), *Phvul.009G256400* (*PR2*), and *Phvul.003G109100* (*PR1a*) in the common bean cultivar of California Dark Red Kidney at 24, 48, 72, 96, and 120 h post-inoculation (hpi) with race 73 of *C. lindemuthianum* and mock. Yellow shading indicates higher expression, and dark blue indicates lower expression.

Regarding the pathogenesis-related genes *PR1b* (*Phvul.006G196900*), *PR2* (*Phvul.009G256400*), and *PR1a* (*Phvul.003G109100*), their reaction to the pathogen was high, with their expression surging by more than 6-fold and, in some cases, even up to 16.7-fold post-inoculation (Figure 4 and Figure 5, and Table 1). Among them, *PR1b* (*Phvul.006G196900*) stood out as the most responsive to the pathogen, exhibiting a substantial 13.6-fold increase in expression from 24 to 96 hpi and a remarkable 16.7-fold increase at 120 hpi (Figure 4 and Figure 5, and Table 1). The gene *PR2* (*Phvul.009G256400*) showed a moderate level of expression and response upon exposure to the pathogen. Remarkably, there was a 10-fold increase in expression observed at both 24 and 48 hpi, alongside an 11-fold increase at 72, 96, and 120 hpi (Figure 4B). Similarly, the gene *PR1a* (*Phvul.003G109100*) exhibited a significant increase from 6.2- to 8.1-fold in expression following inoculation (Figure 4C). These findings shed light on the distinct patterns of gene response to the pathogen, indicating that *Phvul.009G256400* and *Phvul.003G109100* may play crucial roles in plant defenses.

### 2.3. Differential Expression of Candidate and Defense Genes in CDRK Cultivar Inoculated with P. griseola Race 63-39

**Figure 6:**
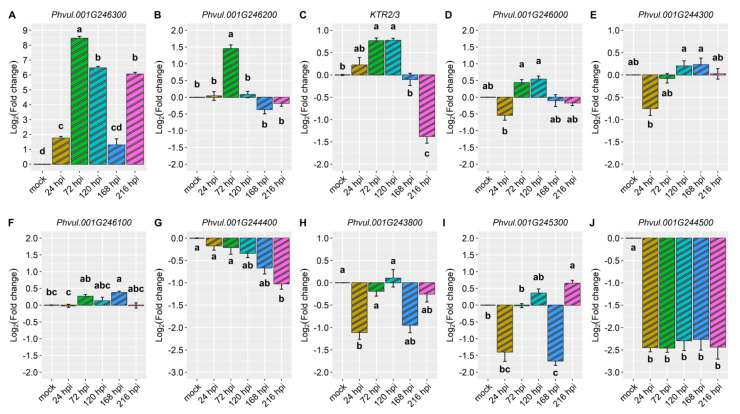
Relative expression of candidate genes: (**A**) *Phvul.001G246300*; (**B**) *Phvul.001G246200*; (**C**) *KTR2/3*; (**D**) *Phvul.001G246000*; (**E**) *Phvul.001G244300*; (**F**) *Phvul.001G246100*; (**G**) *Phvul.001G244400*; (**H**) *Phvul.001G243800*; (**I**) *Phvul.001G245300*; and (**J**) *Phvul.001G244500* in California Dark Red Kidney at 24, 72, 120, 168, and 216 h post-inoculation (hpi) with race 63-39 of *P. griseola* and a mock. The results are presented as logarithmic base 2 of the fold change of gene expression. Means with the same letter, for each gene, are not significantly different at the 5% significance level, using the Alexander-Govern test.

**Figure 7:**
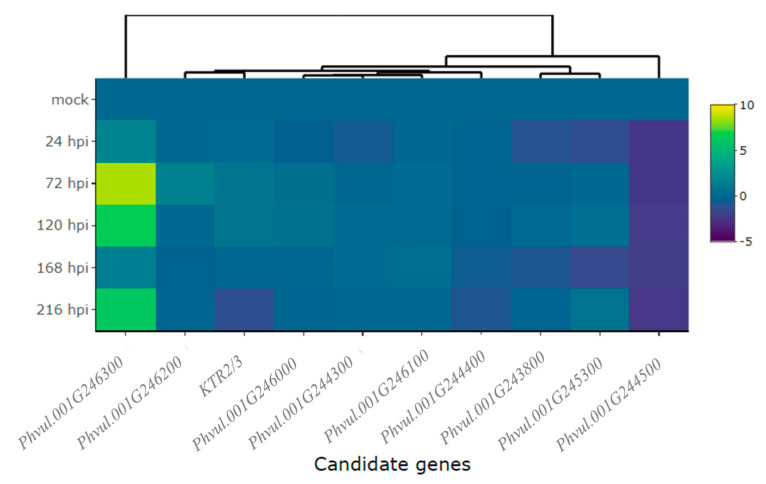
Heatmap of relative expression of candidate genes for the *CoPv01^CDRK^/PhgPv01^CDRK^* and genes proximal to these loci in California Dark Red Kidney at 24, 72, 120, 168, and 216 h post-inoculation (hpi) with race 63-39 of *P. griseola* and a mock. The genes evaluated were *Phvul.001G246300*, *Phvul.001G246200*, *KTR2/3*, *Phvul.001G246000*, *Phvul.001G244300*, *Phvul.001G246100*, *Phvul.001G244400*, *Phvul.001G243800*, *Phvul.001G245300,* and *Phvul.001G244500*. Yellow shading indicates higher expression and dark blue shading lower expression.

Upon inoculating the CDRK cultivar with race 63-39 of *P. griseola*, significant alterations in the relative expression levels of candidate genes for the *CoPv01^CDRK^/PhgPv01^CDRK^* loci and resistance genes were observed. Among the candidate genes assessed at the *CoPv01^CDRK^/PhgPv01^CDRK^* loci, the *Phvul.001G246300* gene showcased the utmost responsiveness to race 63-39 of *P. griseola* in the CDRK cultivar, as illustrated in Figure 6A and Figure 7. Notably, this gene exhibited a substantial increase in expression at 72 hpi, reaching a fold change of up to 8.5-fold (Figure 6A). Furthermore, at 120 and 216 hpi, the gene maintained a consistent mean fold change of 6.1-fold, indicating sustained response to the pathogen. These findings emphasize the potential significance of *Phvul.001G246300* in the response of the CDRK cultivar to *P. griseola* race 63-39.

The gene *Phvul.001G246200* showed induction only at 72 hpi, with an increase of nearly 1.5-fold (Figure 6B). As for the KTR2/3 gene, it exhibited induction at 72 and 120 hpi, with a fold change increase of 0.8, but was subsequently downregulated at 216 hpi (Figure 6C). The gene *Phvul.001G246000* demonstrated minimal changes in expression levels at 72 and 120 hpi (Figure 6D), while *Phvul.001G244300* exhibited similar patterns with few changes at 120 and 168 hpi (Figure 6E). The *Phvul.001G246100* gene displayed induction from 72 to 168 hpi, albeit with a small increase of 0.3-fold (Figure 6F).

On the contrary, the *Phvul.001G244400* gene displayed downregulation at 216 hpi, with a reduction of 1-fold (Figure 6G). The *Phvul.001G243800* gene exhibited downregulation at 24 hpi, with a reduction of 1-fold (Figure 6H). *Phvul.001G245300* demonstrated downregulation at 24 and 168 hpi, followed by induction at 120 and 216 hpi, but with a slight increase of 0.4 and 0.7-fold (Figure 6I). The gene *Phvul.001G244500* consistently showed downregulation at all evaluated time points, with a significant reduction of approximately 2.3-fold in response to the pathogen (Figure 6J). These observations provide valuable insights into the dynamic expression patterns of these genes in response to the pathogen, shedding light on their potential roles in the defense mechanisms of CDRK plants.

**Figure 8:**
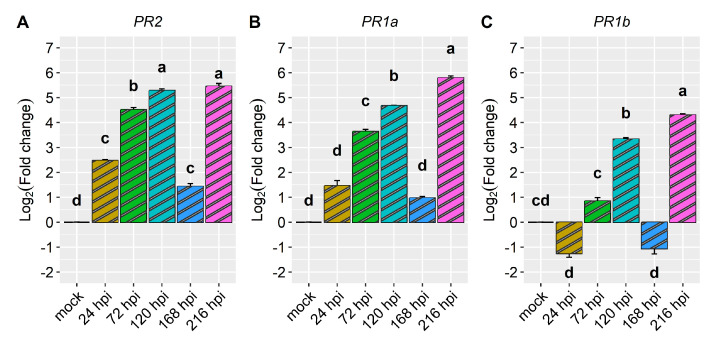
Relative expression of plant defense genes: (**A**) *Phvul.009G256400* (*PR2*)*;* (**B**) *Phvul.003G109100* (*PR1a*); and (**C**) *Phvul.006G196900* (*PR1b*) in the common bean cultivar of California Dark Red Kidney at 24, 72, 120, 168, and 216 h post-inoculation (hpi) with race 63-39 of *P. griseola* and mock. The results are presented as logarithmic base 2 of the fold change of gene expression. Means with the same letter, for each gene, are not significantly different at the 5% significance level, using the Alexander-Govern test.

**Figure 9:**
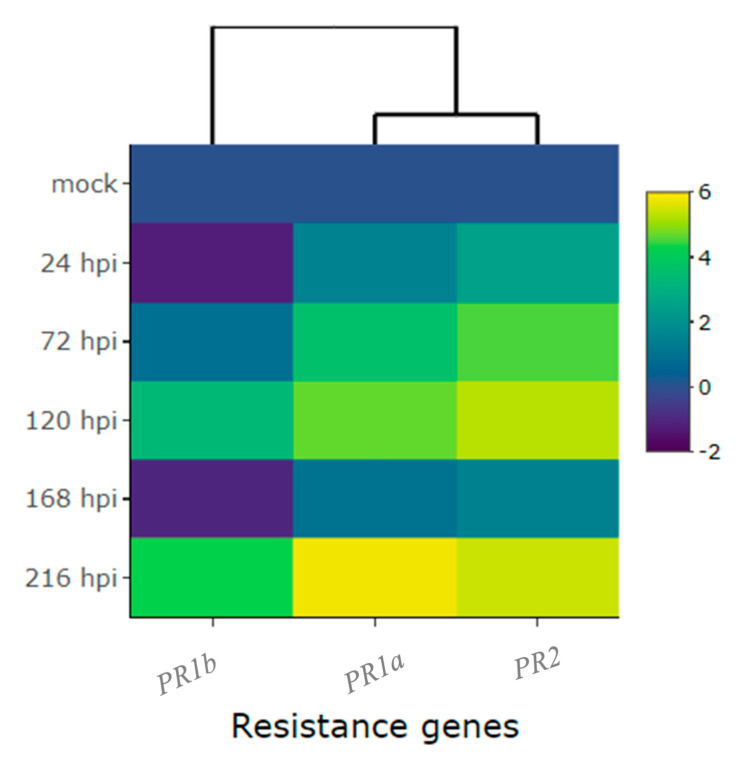
Heatmap of relative expression of plant defense genes *Phvul.006G196900* (*PR1b*), *Phvul.003G109100* (*PR1a*) and *Phvul.009G256400* (*PR2*) in the common bean cultivar of California Dark Red Kidney at 24, 72, 120, 168, and 216 h post-inoculation (hpi) with race 63-39 of *P. griseola* and a mock. Yellow shading indicates higher expression and dark blue has lower expression.

When the CDRK cultivar was inoculated with race 63-39 of *P. griseola*, pathogen-related genes exhibited high expression responses, as evidenced in Figure 8 and Figure 9. The *PR2* gene displayed a significant increase in expression at 72 hpi, reaching peak induction levels at 120 and 216 hpi with fold increases of around 5.3. At 24 and 168 hpi, there was a slight increase in the expression level of this gene, with an average fold change above 1.9 (Figure 8A and Table 1). The *PR1a* gene demonstrated an increase in gene expression starting from 24 hpi and peaking at 216 hpi, with a fold increase of nearly six in response to the pathogen (Figure 8B). The *PR1b* gene exhibited induction at 120 and 216 hpi, with fold changes above three and four, respectively. However, at 24 and 168 hpi, the expression level of this gene decreased compared to 72, 120, and 216 hpi, but no differences were observed compared to the mock (Figure 8C). Overall, the *PR1a* and *PR2* genes showed similar expression patterns and were more responsive to race 63-39 of *P. griseola* than the *PR1b* gene (Figure 9).

## 3. Discussion

This study discusses the cellular mechanisms employed by plants to combat unfavorable conditions caused by biotic factors. These responses are complex networks that involve changes in gene expression, regulation of metabolic processes, reinforcement of the plant cell wall, and hormone signaling pathways. In particular, the majority of resistance genes identified encode NBS-LRR proteins, which consist of an amino-terminal signaling domain, a nucleotide-binding site (NBS), and carboxy-terminal leucine-rich repeats (LRRs). These NBS-LRR proteins are capable of recognizing pathogen effectors through protein–protein interactions, subsequently triggering effector-triggered immunity [25,26]. However, it is noteworthy that the common bean chromosome Pv01 exhibits a low abundance of NBS-LRR genes. Instead, this chromosome harbors genes that encode other proteins involved in the resistance response, such as kinases functioning as pattern-recognition receptors (PRRs) that recognize pathogen-associated molecular patterns (PAMPs) and initiate PAMP-triggered immunity (PTI).

In this research, we assessed the expression levels of candidate genes near the *CoPv01^CDRK^/PhgPv01^CDRK^* loci and plant defense genes in CDRK cultivar. Through this analysis, we identified genes that consistently manifested high expression in resistant plants under pathogen inoculation. We observed that the candidate gene *Phvul.001G246300* as a potential candidate for the *CoPv01^CDRK^/PhgPv01^CDRK^* resistance loci in CDRK plants inoculated with *C. lindemuthianum* race 73, as it demonstrated the highest relative expression among the candidate genes. Among the tested candidate genes, the expression of *Phvul.001G246300* demonstrated a significant 7.8-fold increase at 24, 48, 72, 96, and 120 hpi of race 73 of *C. lindemuthianum*. Moreover, it exhibited an 8.5-fold change at 72 hpi and over a 6-fold change at 120 and 216 hpi with race 63-39 of *P. griseola*, indicating its heightened responsiveness to both pathogens compared to the other candidate genes examined. This particular gene encodes an abscisic acid (ABA) receptor, PYL5 protein, which is known to play a crucial role in mediating the crosstalk between ABA and jasmonic acid (JA) responses [27]. ABA has been implicated in plant defense against pathogens and shows synergistic interactions with the ethylene (ET) signaling pathway. The upregulation of the PYR receptor during biotic stress suggests its involvement in perceiving ABA and initiating downstream signaling mediated by kinases [28].

The *Phvul.001G245300* gene ranked second in terms of induction among the candidate genes for *CoPv01^CDRK^/PhgPv01^CDRK^* loci in plants inoculated with race 73 of *C. lindemuthianum.* However, its expression was only half that of *Phvul.001G246300*, and when the plants were inoculated with race 63-39 of *P. griseola*, this gene showed a less pronounced response, with higher expression observed only at 216 hpi. Hypothetically, this gene encodes a protein belonging to the protein kinase superfamily, which is predominantly composed of catalytic domains of serine/threonine-specific and tyrosine-specific protein kinases. Furthermore, the protein contains a leucine-rich repeat (LRR) domain. Proteins containing LRRs, including tyrosine kinase receptors, are involved in diverse biological processes such as signal transduction, cell adhesion, DNA repair, recombination, transcription, RNA processing, apoptosis, disease resistance, and immune responses [29,30].

The *Phvul.001G246200* gene ranked third among the upregulated genes, mainly between 24 and 72 hpi with race 73 of *C. lindemuthianum*, but its induction was three times lower than that of *Phvul.001G246300*. When plants were inoculated with race 63-39 of *P. griseola*, this gene exhibited a modest upregulation at 72 hpi, five times less than *Phvul.001G246300*. In Arabidopsis thaliana, a homologous gene plays a crucial role in xylan acetylation and the proper deposition of secondary walls [31]. Acetylation of wall polymers is important for cell wall strength and disease resistance, as evidenced by several Arabidopsis mutants and overexpression lines [31,32]. Therefore, the *Phvul.001G246200* gene may contribute to the resistance response in the CDRK cultivar by modifying cell wall strength, thereby impeding pathogen infection and/or colonization of plant tissues during the biotrophic life stage.

**Figure 10:**
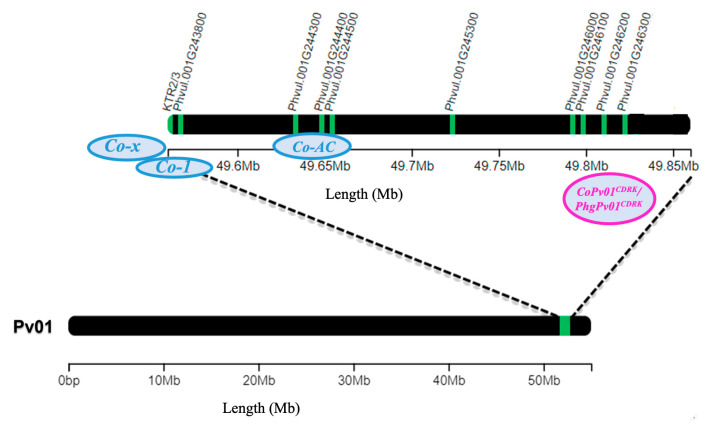
Common bean chromosome Pv01 containing candidate genes for anthracnose resistance genes *Co-x* (*KTR2/3*), *Co-1* (*Phvul.001G243800*), *Co-AC* (*Phvul.001G244300*, *Phvul.001G244400*, and *Phvul.001G244500*), and *CoPv01^CDRK^/PhgPv01^CDRK^* (*Phvul.001G245300*, *Phvul.001G246000*, *Phvul.001G246100*, *Phvul.001G246200,* and *Phvul.001G246300*).

The majority of the ANT resistance genes identified in Andean cultivars are located in a resistance cluster on the common bean chromosome Pv01, including *Co-1*, *Co-1*^2^, *Co-1*^3^, *Co-1*^4^, *Co-1*^5^, *Co-1^HY^*, *Co-1^X^*, *Co-x*, *Co-AC*, and *CoPv01^CDRK^/PhgPv01^CDRK^* (Figure 10). Interestingly, the resistance gene from the Jalo EEP558 cultivar, *Co-x*, conferring resistance against race 3993 of *C. lindemuthianum*, is located in close proximity to *CoPv01^CDRK^/PhgPv01^CDRK^* loci. The protein KTR2/3 has been identified as the controlling factor for resistance of *Co-x* in Jalo EEP558 cultivar [23]. In our study with the CDRK cultivar, we observed that the expression of the *KTR2/3* gene was 2.6 times lower than that of *Phvul.001G246300* at 72 hpi in response to race 73 of *C. lindemuthianum*. Furthermore, in response to race 63-39 of *P. griseola*, the *KTR2/3* gene exhibited a remarkably lower expression level, approximately 10.6 times lower, compared to *Phvul.001G246300*. Taking together, the physical position where *CoPv01^CDRK^/PhgPv01^CDRK^* and *Co-x* were mapped, and different candidate genes significantly upregulated after inoculation in each cultivar, *KTR2/3* for Jalo EEP558 and *Phvul.001G246300* for CDRK, and its possible to conclude that different genetic resistances are involved in each cultivar against the same pathogen.

Using near-isogenic lines with differing resistance alleles, expression analysis of candidate genes for the *Co-1^2^* allele against *C. lindemuthianum* race 73 resistance revealed high levels of *Phvul.001G243800* in the resistant NIL [22]. Furthermore, based on transcriptional analysis, it was observed that *Phvul.001G243700*, located near the *Co-1* locus, was differentially upregulated in the resistant NIL at 72 and 96 hpi after race 73 inoculation [33]. In the present study, significant differences in the expression of the *Phvul.001G246300* and *Phvul.001G243800* genes in response to race 73 of *C. lindemuthianum* and race 63-39 of *P. griseola* were observed in CDRK cultivar. At 72 hpi with race 73 of *C. lindemuthianum,* we observed a notable expression of the *Phvul.001G246300* gene, which was approximately three times higher compared to *Phvul.001G243800*. Remarkably, within the context of *P. griseola* race 63-39, we observed that *Phvul.001G243800* gene was repressed, and on the other hand, the expression of *Phvul.001G246300* gene exhibited a substantial increase of more than 8-fold.

Overall, our findings underscore the contrasting expression patterns of the *Phvul.001G246300* and *Phvul.001G243800* genes within the CDRK cultivar, shedding explanation on their potential roles in the defense mechanisms against *C. lindemuthianum* race 73 and *P. griseola* race 63-39. It is worth noting that the *Co-1* and *CoPv01^CDRK^/PhgPv01^CDRK^* loci were accurately mapped to separate regions towards the terminal end of the common bean chromosome Pv01, positioned 211 kb apart. Consequently, it is of paramount importance to emphasize that the CDRK cultivar harbors a distinct and independent gene from the *Co-1* locus.

The pathogenesis-related defense genes *PR1a*, *PR1b*, and *PR2* exhibited significant responsiveness to the pathogen. Notably, *PR1b* displayed the highest level of responsiveness to pathogen race 73, with a pronounced increase in expression at 120 hpi, reaching up to 16.7-fold higher than the control. Both *PR2* and *PR1a* displayed elevated expression levels, exhibiting 11.4-fold and 8.1-fold increases, respectively, compared to the mock. Notably, *PR2* maintained consistently high expression levels from 24 hpi to 120 hpi in response to the pathogen. Mahiya-Farooq et al. [22] reported early expression of plant defense genes in the resistant NIL, with *PR1b* and *PR2* showing accumulation at 24 hpi of *C. lindemuthianum* race 73. Similarly, Shams et al. [34] observed higher expression of *PR2* in the Naz-resistant bean cultivar upon inoculation with *C. lindemuthianum* race 2. Although to a lesser extent, these pathogenesis-related defense genes were also upregulated in plants inoculated with race 63-39 of *P. griseola*. *PR1a* and *PR1b* showed higher induction at 216 hpi, with nearly a 6-fold increase and over a 4-fold increase in gene expression, respectively. The *PR2* gene exhibited a more than 5-fold increase at 120 and 216 hpi. Interestingly, at 168 hpi, all the genes displayed reduced or no responsiveness to the pathogen.

These results reveal that the ANT and ALS resistance genes in the CDRK cultivar are controlled independently from those previously identified at the *Co-1* locus. This indicates that the robust resistance against ANT and ALS in CDRK is manifested through the heightened response of the candidate gene *Phvul.001G246300* to the respective pathogens. These findings point out the complex nature of plant–pathogen interactions, emphasizing the significance of comprehending gene expression mechanisms. Our findings might contribute to an enhanced comprehension of novel and efficient strategies for the development of cultivars resistant to angular leaf spot and ANT.

## 4. Materials and Methods

### 4.1. Plant Material, Growth Conditions, and Experimental Design

Two experiments were conducted using a completely randomized design. In Experiment I, seedlings from both the resistant (R) CDRK and the susceptible (S) Yolano cultivars were inoculated with race 73 of *C. lindemuthianum*. The isoline derived from the CDRK × Yolano cross, namely, CY 19, was used as a positive control for disease scoring to ensure that the fungus was virulent on them and that the absence of disease on the resistant genotypes was not attributed to the fungus lacking virulence. Additionally, the CY 70 isoline was used as a resistant control. The relative expression levels of 13 specific genes only in CDRK cultivar were evaluated at multiple time points: 24, 48, 72, 96, and 120 h post-inoculation (hpi), as well as in the mock. In Experiment II, a parallel approach was adopted, involving the same genotypes as in Experiment I. However, the genotypes were subjected to inoculation with race 63-39 of *P. griseola*. Similarly, the relative expression of the same 13 genes only in the CDRK cultivar were evaluated at 24, 72, 120, 168, and 216 hpi, in addition to the mock condition. Each experimental condition was replicated across three separate biological replicates (plants). Within each biological replicate, the assessment was further reinforced by performing three technical replicates of quantitative polymerase chain reaction (qPCR) for each experiment.

The experiments were conducted at the Núcleo de Pesquisa Aplicada à Agricultura (Nupagri) at the Universidade Estadual de Maringá (UEM) in Maringá, Paraná, Brazil (latitude 23° 26′8″ S, longitude 51° 53′42″ W). Briefly, seeds were planted in plastic trays filled with a commercial substrate, MecPlant (MEC PREC—Ind. Com Ltd.a, Telemaco Borba, Brazil), that had been previously sterilized and fertilized. The seedlings were grown in greenhouses under natural light at a temperature of 25 °C until the first trifoliate leaf growth stage [35].

### 4.2. Pathogenesis Assays

Monosporic cultures of *C. lindemuthianum* and *P. griseola* were prepared according to the methodologies described by Mathur et al. [36] and Sanglard et al. [37], respectively. Inocula of the ANT pathogen races were produced by incubating them on a young green common bean pod medium [38] at 22 °C for 14 days. The inoculum for the ALS pathogens was multiplied in Petri dishes with tomato medium [37,39] containing 1.61% agar (*m*/*v*), 0.25% calcium carbonate (*m*/*v*), 61.94% distilled water (*v*/*v*), and 36.2% V8^®^ vegetable juice (Campbell’s company soup (*v*/*v*)) and maintained in a bio-oxygen demand incubator at 24 °C for 18 days. The concentration of fungal spores for the ANT pathogen was adjusted to 1.2 × 10^6^ [38]. For the ALS pathogen, it was adjusted to 1.2 × 10^4^ conidia ml^−1^ [40] using a hemacytometer (1/400 mm^2^, Hausser Scientific, Horsham, PA, USA). Fourteen-day-old seedlings were inoculated with each race of the pathogen on the underside of their leaves. The inoculation process was carried out by using a manual pressurized pump sprayer for spraying. For the mock treatment, which served as the negative control, the seedlings were sprayed with only distilled water and Tween 20^®^ (0.01%). After inoculation, the plants were maintained in a mist chamber at >95% relative humidity, at a temperature of 20 ± 2 °C, with 12 h of daily light (680 lux) for 72. Following the inoculation process, the seedlings were moved to benches and placed under the same conditions as before, except for a high-humidity environment. This environment was maintained until the end of the experiment when all the samples were collected. Anthracnose and angular leaf spot symptoms were evaluated using 1-to-9 disease severity scales proposed by Pastor-Corrales et al. [35] and Inglis et al. [41]. Plants with disease reaction scores between 1 and 3 were considered resistant, whereas plants with scores from 4 to 9 were considered susceptible.

### 4.3. Total RNA Extraction and cDNA Synthesis

Leaf samples were collected from CDRK plants before inoculation (mock) and during incompatible reactions with race 73 of *C. lindemuthianum*, as well as with race 63-39 of *P. griseola*. Sampling was performed at specific time points critical for pathogens development: 24, 48, 72, 96, and 120 hpi for *C. lindemuthianum*, and 24, 72, 120, 168, and 216 hpi for *P. griseola*. Samples were obtained from three biological replicates for each pathogen. To ensure RNA integrity, the leaf samples were promptly frozen in liquid nitrogen for subsequent extraction [42].

Total RNA was extracted from 100 mg frozen and purified using GeneJet Plant RNA Purification Kit (Thermo Fisher Scientific, Waltham, MA, USA) following the manufacturer’s instructions. The integrity of the total RNA was assessed with electrophoresis on a 1% m/v agarose gel, run for 80 min at 80 volts, at 5 °C, and in the absence of light. For the assessment of both the quality and quantity of total RNA, a spectrophotometer (FEMTO 700S^TM^) was employed to measure absorbance at specific wavelengths: 230 nm, 240 nm, 260 nm, and 280 nm. The criteria for RNA purity were determined based on the following absorbance ratios: A_260_/A_230_ ranging from 1.9 to 2.4, A_260_/A_240_ of at least 1.4, and A_260_/A_280_ between 1.8 and 2.2. To compute the concentration of total RNA, the formula [RNA] (ng µL^−1^) = A_260 nm_ × 40 × 100, as outlined by Farrell [43], was utilized. Total RNA samples that met the purity and integrity criteria were treated with DNase I^TM^ (Invitrogen™, Waltham, MA, USA) to eliminate any possible genomic DNA contamination. The purification reaction involved 1 µg of total RNA, following the manufacturer’s instructions.

To synthesize cDNA, the “Superscript^®^ IV First-Strand Synthesis System” kit (Invitrogen™, Waltham, MA, USA) was used according to the manufacturer’s instructions. The cDNA synthesis reaction was prepared with a total volume of 20 µL, containing the following constituents: 1 µg of total RNA, primer-oligo d(T) at a concentration of 2.5 µM, dNTP mix with each nucleotide at 0.5 mM, First-Strand Buffer at 1× concentration, DL-dithiothreitol at 5 mM, ribonuclease inhibitor with a concentration of 2 U µL^−1^, MMLV-RT at 10 U µL^−1^, and RNase-free water. The procedure was initiated by combining total RNA, primer-oligo d(T), dNTP mix, and RNase-free water to reach a cumulative volume of 13 µL. The samples were incubated in a thermocycler (Applied Biosystems^®^ Veriti^®^ 96-Well Fast Thermal Cycler, Waltham, MA, USA) at 65 °C for 5 min, followed by 4 °C for 1 min. Then, the First-Strand Buffer, DL-dithiothreitol, ribonuclease inhibitor, and 1 MMLV-RT were added to the reaction. The samples were incubated at 55 °C for 10 min for cDNA synthesis activation, followed by 80 °C for 10 min to inactivate the reaction. To remove residual RNA after cDNA synthesis, 1 µL of Escherichia coli RNase H was added, and the samples were incubated at 37 °C for 20 min. The cDNA synthesis product (20 µL) was diluted 1:100 for qPCR analysis. To assess the cDNA synthesis efficiency, positive control was included, in which HeLa-S3 RNA (10 ng) was used instead of total RNA.

To verify the quality of the cDNA synthesis, a PCR reaction was performed on the positive control and a negative control (containing water instead of cDNA) using the following reaction mix: 5 µL PCR buffer (10×), 2 µL MgCl (50 mM), 1 µL dNTP Mix (10 mM), 1 µL sense primer (10 µM), 1 µL antisense primer (10 µM), 2 µL of cDNA for the positive control, and 2 µL of ultrapure H_2_O for the negative control, 0.2 µL of Taq PlatinumTM DNA polymerase (Invitrogen™, Waltham, USA), and 37.8 µL of ultrapure H_2_O. The PCR reaction was performed for 35 cycles, with an initial denaturation at 94 °C for 2 min, denaturation at 94 °C for 15 s, annealing at 55 °C for 30 s, and synthesis at 68 °C for 1 min. The PCR products were analyzed using electrophoresis on a 1.5% *m*/*v* agarose gel. The positive control showed a single band of approximately 353 bp, while no product was observed in the negative control, confirming the efficiency of the cDNA synthesis.

### 4.4. Target Genes and Primer Design

Ten candidate genes were selected for expression analysis in CDRK plants inoculated with race 73 of *C. lindemuthianum* and race 63-39 of *P. griseola*. The genes evaluated in the *CoPv01^CDRK^*/*PhgPv01^CDRK^* resistance loci were *Phvul.001G246000*, *Phvul.001G246100*, *Phvul.001G246200*, and *Phvul.001G246300*. Additionally, the gene *Phvul.001G245300* located near these loci was also assessed [15]. The genes proposed for the *Co-AC* locus, namely, *Phvul.001G244300*, *Phvul.001G244400*, and *Phvul.001G244500* [14], were also tested. The gene *Phvul.001G243800* was evaluated as it was induced in the near isogenic line T9576R, which carries the *Co-1^2^* resistance allele, when inoculated with race 73 of *C. lindemuthianum* [22]. The *KTR2/3* gene, a candidate gene for *Co-x* in Jalo EEP558, which was induced in response to strain 100 of *C. lindemuthianum*, was also included [23]. Finally, three known plant defense genes, namely, *Phvul.003G109100* (*PR1a*), *Phvul.006G196900* (*PR1b*), and *Phvul.009G256400* (*PR2*), were evaluated [16,22,42]. To standardize gene expression levels, we employed the reference genes *Phvul.008G011000* (actin–ACT) and *Phvul.001G133200* (insulin-degrading enzyme–IDE) [44]. ACT had previously been validated for quantifying the relative expression of candidate genes in studies conducted by Mahiya-Farooq et al. [22] and Shams et al. [34], while IDE’s validation was previously established by Oblessuc et al. [20]. Both genes were utilized as reference genes for quantifying the relative expression of resistance genes against ANT in studies conducted by Borges et al. [44] and Richard et al. [23].

To design primers for qPCR, the coding sequences (CDS) and DNA sequences of the target genes were downloaded from the common bean (*P. vulgaris* L.) genome available at Phytozome 12 [45]. The “Primer-BLAST web tool” [46] was used to design primers that met the following specifications for efficient qPCR: primer size 18-24 bp, melting temperature between 59–61 °C, amplicon size between 80-160 bp, and whenever possible, at least one intron on the corresponding genomic DNA sequence was included between the primer pair. The primers for the *KTR2/3* gene were obtained from Richard et al. [23].

To ensure the specificity and efficiency of the primers, dimers and secondary structures were checked using Gene Runner software (version 6.5.52), the “Multiple Prime Analyzer” web tool (Thermo Fisher Scientific: https://bit.ly/34kZpnP (accessed on 29 May 2020)), and “The Sequence Manipulation Suite” web tool [47]. The amplicon secondary structure was also verified using the “The Mfold Web Server” platform [48] with coding sequences downloaded from Phytozome 12. All procedures used for primer design and in silico validation followed literature recommendations [49,50]. The primer sequences for each candidate gene evaluated are listed in Table 2.

### 4.5. Quantitative PCR (qPCR) and Data Analysis

The determination of PCR efficiency for each primer involved establishing a standard curve through a fivefold serial dilution, utilizing the cDNA pool as the template. This process incorporated three replicates at every dilution point, following the methodologies outlined by Svec et al. [51] and Rasmussen [52]. The amplification efficiency was computed employing the equation E = [10^(−1/slope^)] − 1 [52], utilizing the slope values derived from linear regression analysis. This analysis encompassed the log_10_-transformed cDNA concentrations on the x-axis and corresponding Cq values on the y-axis. The calculated amplification efficiency for each primer pair ranged from 0.92 to 1.09, while maintaining a coefficient of determination (R^2^) for the linear regression of at least 0.97 (Table 2).

**Table 2:** Target genes, primers used, qPCR product size (amplicon), primer melting temperature (Tm), amplification efficiency (E,) and coefficient of determination of linear regression (R²).

Gene Model ^a^	Reference Genes	Primers Forward (F) and Reverse (R) (5′-3′)	Amplicon (bp)	Tm (°C)	E ^b^	R² ^c^
*Phvul.001G133200*	*IDE*	F: AAGCAGGTATCTTGGCCATCTC	126	60.16	1.04	0.99
		R: AAAGCAAACTCCAAGCTCCAATC		59.99		
*Phvul.008G011000*	*ACT*	F: GAAGTTCTCTTCCAACCATCC	154	59.67	1	0.98
		R: TTTCCT TGCTCATTCTGTCCG		58.38		
*Phvul.001G243800*	*Co-1*	F: CCTCAAGGTGGGGCTTTTGAG	118	61.16	1.04	0.99
		R: TCACCGAGAAACTCCCATTGC		60.61		
*KTR2/3*	*Co-x*	F: ATGCACAGGGGAATGGGATG	279	60.11	1.04	0.98
		R: GCCATAGCGAGTGAGAGTGCG		63.42		
*Phvul.001G244300*	*Co-AC*	F: GAAACGTCTCCGCAGAATAGTG	150	59.4	1.03	0.99
		R: GTCTTGTGTTGTTCCTTGGAGTTG		60.44		
*Phvul.001G244400*	*Co-AC*	F: TACAGCAAGAGAGCGGTTAAAGG	121	60.62	1.01	0.99
		R: CCCTTTGTCACTTTGTTTTGAAGC		59.67		
*Phvul.001G244500*	*Co-AC*	F: CAATGCACAGCTCGCAACTC	141	60.45	1.09	0.97
		R: GGAACTGTGAAAGCTCTGCTAAC		59.81		
*Phvul.001G245300*	*CoPv01^CDRK^/* *PhgPv01^CDRK^*	F: TCTGCTGGAAGGGTGGTAGTC	93	61.17	1.07	0.98
		R: GGACGTTATGTGAACAAGGTTTGC		61.08		
*Phvul.001G246000*	*CoPv01^CDRK^/* *PhgPv01^CDRK^*	F: ATGAAGCGGATGGATGTCTTG	132	58.43	1.01	0.97
		R: TCTACGAAGCTTAGGCAATTGAG		58.57		
*Phvul.001G246100*	*CoPv01^CDRK^/ PhgPv01^CDRK^*	F: CACGGTATCCTCAGCGAAGAC	119	60.53	1.05	0.99
		R: CAGCAGTCAGCACATACTGGAG		60.99		
*Phvul.001G246200*	*CoPv01^CDRK^/ PhgPv01^CDRK^*	F: GAAGGAGGCTGTGACGTGTTC	150	61.2	1.04	0.99
		R: CCATCGCCACCGTTGATACTC		61.13		
*Phvul.001G246300*	*CoPv01^CDRK^/ PhgPv01^CDRK^*	F: CTTCTTCCCTTCACTTCGATACC	87	58.57	1.09	0.99
		R: GTTGAGAGTGTTTGTGGCAGT		58.98		
*Phvul.003G109100*	*PR1a*	F: GTCCTAACGGAGGATCACTCA	148	58.62	1.06	0.99
		R: CAGGGATTGGCCAGAAGGTAT		59.5		
*Phvul.006G196900*	*PR1b*	F: GGTTTGCCTATGATCCCAATGC	115	59.96	1.03	0.99
		R: TGTTGTGAGCGTTGAGGAAGTC		61.06		
*Phvul.009G256400*	*PR2*	F: CAGAGGTTCTCATTTGCTGCTTTC	98	60.62	1.07	0.99
		R: ATGCCATAACACACCCCGATTTG		61.75		

^a^ Based on the *Phaseolus vulgaris* genome available on the Phytozome 12 platform: https://phytozome.jgi.doe.gov/pz/portal.html# (accessed on 29 May 2020). ^b^ Amplification efficiency obtained from Equation E = [10^(−1/slope)^] − 1 [52]. ^c^ Coefficient of determination of linear regression.

The cDNA quantification reactions were conducted in the StepOnePlus™ real-time PCR system (Applied Biosystems™; StepOnePlus™ Real-Time PCR Systems) using 96-well microplates (MicroAmp™ Fast 96 -well Reaction Plate (0.1 mL)) sealed with MicroAmp™ Optical Adhesive Film. The total reaction volume was 10 µL, consisting of 3.4 µL of cDNA, 1.6 µL of forward and reverse primer mix (800 nM), and 5 µL of PowerUp™ SYBR™ Green Master Mix (Applied Biosystems™, Waltham, MA, USA). The thermocycling conditions included 50 °C for 2 min, 95 °C for 2 min, 40 cycles of 15 s at 95 °C, and 30 s at 60 °C.

After completion of the cDNA quantitation reaction, a dissociation curve was performed to verify target specificity using the manufacturer’s standard continuous melt curve setup, and only samples exhibiting specificity based on the dissociation curve were used. Cq (quantification cycle) values were obtained using StepOnePlus™ Software v2.3 (Applied Biosystems™), with the baseline determined automatically and the threshold was determined manually in the exponential phase of amplification (0.7707 for all cDNA quantification reactions).

The genes *Phvul.001G133200* (*ACT*) and *Phvul.008G011000* (*IDE*) were used as reference genes [44], with the arithmetic mean of C_T_ values [53] calculated in each experimental condition evaluated. Relative expression levels were determined as follows by normalizing the C_T_ values with reference genes using the 2^−ΔΔCT^ method [54,55]:

ΔΔC_T_ = [(C_T_ gene of interest − arithmetic mean of C_T_ of reference genes) at time x − (C_T_ gene of interest − arithmetic mean of C_T_ of reference genes) at mock].

The mean C_T_ values for each gene under each experimental condition were calculated based on three biological replicates and three technical replicates (n = 3 × 3).

The relative expression of candidate genes to the *CoPv01^CDRK^*/*PhgPv01^CDRK^* loci and known disease resistance genes were investigated in response to race 73 of *C. lindemuthianum* at 24, 48, 72, 96, and 120 hpi (Experiment I), as well as in response to race 63-39 of *P. griseola* at 24, 72, 120, 168, and 216 hpi (Experiment II) in CDRK cultivar. The calibrator condition for each gene was the relative expression in the mock (control, without pathogen). To analyze the data and show results, logarithmic base 2 transformation of ΔΔC_T_ values were performed before statistical analysis. The expression levels among experimental conditions were compared using the Alexander-Govern test with a significance level of 5%. Pairwise comparisons of relative expression mean among time points for each gene were assessed, and the significance level was adjusted using Bonferroni correction (*p* ≤ 0.05). These statistical analyses were performed using the “oneway-test” [56] and “companion” R packages. All data wrangling and statistical analysis were performed using R software (version 4.0.3) (R Core Team) [57], with plots generated using the package ggplot2 [58] and R base. Error bars represent the standard deviation of the means from three biological and three technical replicates (3 × 3). Heatmaps were generated with mean C_T_ values using the “heatmaply” R package, and the dendrogram was based on the Euclidean distance measure and the average linkage function [59] among the relative expression of the genes.

## 5. Conclusions

In conclusion, our study provides valuable insights into the genomic organization of ANT and ALS resistance genes on Pv01, specifically in the context of the CDRK cultivar. We highlight the importance of closely situated candidate genes within resistance gene clusters, as they play a crucial role in conferring effective resistance against ANT and ALS pathogens. Furthermore, the *CoPv01^CDRK^* and *PhgPv01^CDRK^* resistance loci are concomitantly inherited and can be efficiently tracked using molecular markers. Our findings shed light on the fact that the *Phvul.001G246300* gene develops a pleiotropic mechanism, demonstrating the highest responsiveness to both pathogens: *C. lindemuthianum* race 73 and *P. griseola* race 63-39. These discoveries carry significant practical implications for breeding endeavors aimed at developing bean cultivars resistant to ANT and ALS, facilitated by the implementation of marker-assisted selection.

## Figures and Tables

**Table 1:** Summary table of mean relative gene expression (Log_2_(fold change)) of *CoPv01^CDRK^*/*PhgPv01^CDRK^* candidate genes and pathogenesis-related genes in response to ANT and ALS in CDRK cultivar.

Gene	Gene Model	*C. lindemuthianum* Race 73 (ANT)					*P. griseola* Race 63-39 (ALS)				
		24 hpi	48 hpi	72 hpi	96 hpi	120 hpi	24 hpi	72 hpi	120 hpi	168 hpi	216 hpi
*Co-x*	*KTR2/3*	1.4	1.3	3.1	0.5	0.5	0.2	0.8	0.8	−0.1	−1.4
*Co-1*	*Phvul.001G243800*	1.4	0.9	2.0	1.8	0.6	−1.1	−0.2	0.1	−0.9	−0.3
*Co-AC*	*Phvul.001G244300*	−0.2	−0.9	−0.2	−1.4	−1.3	−0.8	−0.1	0.2	0.2	0.0
	*Phvul.001G244400*	0.0	0.0	−0.7	−0.4	−0.2	−0.2	−0.2	−0.3	−0.7	−1.0
	*Phvul.001G244500*	0.4	0.3	−0.4	−0.5	0.8	−2.4	−2.5	−2.3	−2.3	−2.4
*CoPv01^CDRK^ /PhgPv01^CDRK^*	*Phvul.001G245300*	3.7	2.5	3.4	3.3	3.5	−1.4	0.0	0.4	−1.7	0.7
	*Phvul.001G246000*	0.9	0.8	0.8	0.7	0.3	−0.5	0.4	0.5	−0.1	−0.2
	*Phvul.001G246100*	0.6	1.5	1.2	0.7	1.3	0.0	0.3	0.1	0.4	0.0
	*Phvul.001G246200*	2.7	2.6	2.6	0.5	1.6	0.0	1.5	0.1	−0.4	−0.2
	*Phvul.001G246300*	7.2	7.3	7.8	7.4	6.3	1.7	8.5	6.5	1.3	6.1
Pathogenesis-related genes	*PR1a*	6.6	6.2	8.0	8.1	6.3	1.5	3.6	4.7	1.0	5.8
	*PR1b*	13.6	13.9	14.1	14.2	16.7	−1.3	0.9	3.3	−1.1	4.3
	*PR2*	10.2	10.1	11.3	11.4	11.0	2.5	4.5	5.3	1.4	5.5

## Data Availability

Data sharing not applicable to this article.

## Supplementary Materials

The supporting information can be downloaded at: https://www.mdpi.com/article/10.3390/ijms242216023/s1.
