# Supplementary material for: Responsiveness of Candidate Genes on CoPv01CDRK/PhgPv01CDRK Loci in Common Bean Challenged by Anthracnose and Angular Leaf Spot Pathogens
Source: Int J Mol Sci. 2023 Nov 7;24(22):16023. doi: 10.3390/ijms242216023 (PMC10671028; doi:10.3390/ijms242216023)
Supplement: Supplementary file 1 [file ijms-24-16023-s001.zip › ijms-2611618-supplementary.pdf]

Table S1. Gene model, previously mapped genes and functional annotation using Phytozome.

**Gene model**	**Gene**	**Functional annotation on Phytozome**
Phvul.001G243800	*Co-1*	Serine/Threonine-protein kinase-like protein CCR3- related
KTR2/3	*Co-x*	Serine/Threonine-protein kinase-like protein CCR3- related
Phvul.001G244300	*Co-AC*	Clathrin Heavy Chain (CLTC) involved in plant defense signaling
Phvul.001G244400	*Co-AC*	Unknown function
Phvul.001G244500	*Co-AC*	Helix-loop-helix DNA-binding domain with possible transcription function
Phvul.001G245300	$CoPv01^{CDRK}$/ $PhgPv01^{CDRK}$	Protein tyrosine kinase (pkinase_tyr) //leucine-rich repeat n-terminal domain (lrrnt_2)
Phvul.001G246000	$CoPv01^{CDRK}$/ $PhgPv01^{CDRK}$	ATP-dependent RNA helicase ddx55/spb4 [ec:3.6.4.13] (ddx55, spb4)
Phvul.001G246100	$CoPv01^{CDRK}$/ $PhgPv01^{CDRK}$	Cation-dependent mannose-6-phosphate receptor
Phvul.001G246200	$CoPv01^{CDRK}$/ $PhgPv01^{CDRK}$	Protein trichome birefringence-like 33
Phvul.001G246300	$CoPv01^{CDRK}$/ $PhgPv01^{CDRK}$	Abscisic acid receptor pyl5
Phvul.003G109100	*PR1a*	Pathogenesis-related protein Bet v I family
Phvul.006G196900	*PR1b*	Pathogenesis-related protein 1
Phvul.009G256400	*PR2*	Pathogenesis-related protein 2
